# Advancing care coordination to enhance shared care for complex cancer survivors in primary care (ACCESS-PC): protocol for a hybrid type 3 effectiveness-implementation trial

**DOI:** 10.1186/s12875-026-03484-2

**Published:** 2026-07-22

**Authors:** Sarah J. Fadem, Benjamin F. Crabtree, Jennifer R. Hemler, Kulwinder Dhaliwal, Pamela Ohman-Strickland, Jeanne M. Ferrante, Shawna V. Hudson, Denalee M. O’Malley

**Affiliations:** 1 Department of Family Medicine and Community Health, Research Division, Rutgers Robert Wood Johnson Medical School, New Brunswick, NJ USA; 2 Rutgers Cancer Institute of New Jersey, New Brunswick, NJ USA; 3 Department of Pediatrics, Rutgers Robert Wood Johnson Medical School, New Brunswick, NJ USA; 4 School of Public Health, Rutgers University, New Brunswick, NJ USA; 5 Institute for Health, Health Care Policy and Aging Research, Rutgers University, New Brunswick, NJ USA

**Keywords:** Cancer survivorship, Primary care, Care coordination, Breast cancer, Prostate cancer, Bladder cancer, Endometrial cancer, Cardiovascular disease, Multimorbidity, Implementation strategies

## Abstract

**Background:**

Cancer survivors with chronic conditions (e.g., complex cancer survivors) are a growing population that faces greater cancer-specific and cardiovascular disease (CVD) related morbidity and mortality. A shared care model, which involves primary care and cancer clinicians, is the optimal care delivery model for complex cancer survivors. Yet, there is little guidance on how to effectively implement and support complex cancer survivors’ continued engagement with primary care during the cancer treatment phase.

**Methods:**

This protocol describes a multi-phase, mixed methods, stakeholder-engaged research process that tailors and tests a multi-level health system intervention to improve primary care provider and cancer team ‘shared care’ for complex cancer survivors. This study employs a design-for-dissemination, theory-guided perspective, blending implementation science and care delivery conceptual frameworks: Exploration, Planning, Implementation, and Sustainment (EPIS) and Cancer Multi-team System (cMTS). These frameworks guide our understanding and strategies deployed on the multi-level factors of implementing shared care in a health system. Primary Care Connect (PCC) is a health system intervention designed to align complex cancer survivors, healthcare team members, and health system implementation actors’ understandings, capacities, and practices to promote the adoption of shared care delivery models for complex cancer survivors. A hybrid type 3 effectiveness-implementation study design will test the effectiveness of PCC (*n* = 266 patients) in a patient-level randomized controlled trial on primary care connection, chronic disease management, and patient-reported outcomes. PCC implementation (e.g., feasibility, acceptability, and appropriateness) will be evaluated using a mixed methods approach to inform sustainable usage of the intervention. A concurrent learning evaluation will assess PCC’s adaptations over time and convene health system stakeholders to explore intervention properties to inform adoption and sustainment considerations.

**Discussion:**

The overarching goal of this protocol is to examine whether cancer survivors becoming “lost in transition” is a preventable problem. PCC tests whether the challenge of transitioning patients back to primary care can be avoided altogether by re-engineering care coordination with primary care at the outset of cancer treatment. Study findings are poised to have a profound impact on the adoption of shared care delivery models throughout the U.S. to optimally mitigate complex cancer survivors’ CVD risks.

**Trial registration:**

Registered with ClinicalTrials.gov on February 19, 2025: NCT06834204.

**Supplementary Information:**

The online version contains supplementary material available at https://doi.org/10.1186/s12875-026-03484-2.

## Background

Enhancing cardiovascular disease (CVD) risk management for cancer survivors is imperative to improving care quality and addressing care disparities. A majority of cancer survivors (70%) have at least one CVD risk factor and greater CVD mortality independent of traditional CVD risk factors [1–8].

The population of cancer survivors with chronic diseases, complex cancer survivors, has grown substantially from 2002 to 2018, with increases between 50 and 200% for almost every chronic condition [9]. These complex cancer survivors are more likely to be diagnosed later [10–12], receive non-curative cancer treatment regimens [13–18], and suffer greater morbidity and mortality [19–22] than survivors with minimal comorbidity. They are also disproportionately represented among priority health disparity populations (i.e., racial/ethnic minorities, low socioeconomic status) [9, 23]. Despite having multiple chronic conditions, many complex cancer survivors underutilize necessary chronic disease care, especially during cancer treatment [24, 25].

CVD burden is not shared equally among cancer survivors—most CVD deaths (61%) occur in patients with history of breast, prostate, or bladder cancer [6], and endometrial cancer survivors have the greatest CVD mortality (vs. all other cancer sites) at all times following cancer diagnosis [5, 26]. This is significant, as breast, prostate, bladder and endometrial cancer account for 50% of the cancer survivors in the United States [27]. CVD mortality risks are highest within the first-year post-cancer diagnosis and remains elevated post-treatment [5, 6]. Poor glycemic control and hypertension management during cancer treatment and survivorship negatively impact survival [15, 27–31].

Shared care is a care delivery model that integrates the delivery of services within and across health systems and enhances communication between teams of providers who share a patient in common [32]. For cancer care, this typically includes primary care, oncology and, when necessary, medical subspecialists [33]. Shared care has been associated with optimal comorbidity management, especially when there is primary care involvement [34–36]. Studies have demonstrated superior outcomes for shared care models for coordination, patient outcomes, symptom management, treatment initiation and medication adherence [33–39]. Previous efforts (e.g., survivorship care plan development) have demonstrated that implementing tools to improve cancer and primary care communication is complicated [40–43]. A landmark AHRQ report synthesizing 75 systematic care coordination reviews across diseases revealed that team-based care was one of the strategies with compelling evidence for improved outcomes [44–46]. Yet, studies testing factors that contribute to effective team-based care for cancer survivors managing comorbidities are notably absent from the extant literature [36].

Most cancer centers are embedded in larger health systems; however, few employ multi-level care coordination strategies to educate patients and prepare collaborating primary care providers (PCPs) for the potential impact of cancer treatments on chronic disease management during and after treatments. Complicating this, in the U.S., adult patients may receive care for their cancer and comorbidities within or across multiple institutions. Additional fragmentation risks emerge when care is provided across multiple institutions, given that these may lack interoperable electronic health records (EHRs), which foster information sharing and facilitate communication [47–49]. In a study of black breast cancer survivors with comorbidities, the majority received primary and cancer care in different systems [50]. By leveraging the communities of practice during the planning phases of the Advancing Care Coordination to Enhance Shared Care for Complex Cancer Survivors in Primary Care (ACCESS-PC) trial, including non-affiliate primary care physicians’ perspectives, we take an explicit quality-focused design-for-dissemination approach that accounts for these care delivery complexities.

Primary Care Connect (PCC), includes multi-level implementation strategies in a health system context to build primary and oncology team capacity to improve chronic disease management during treatment and early post-treatment. The primary goal of PCC is to prevent disconnection from primary care from the outset of a cancer diagnosis for patients managing chronic condition(s) that impact cardiovascular health. PCC standardizes care planning communication protocols between care teams, trains cancer team members, and primes complex cancer survivors with information about the need for shared care beginning at cancer diagnosis. Using a hybrid type 3 effectiveness-implementation study design, we will test PCC’s effectiveness and implementation impact on primary care connectedness, chronic disease management, and patient-reported outcomes [51].

### Theoretical framework and approach

**Fig. 1:**
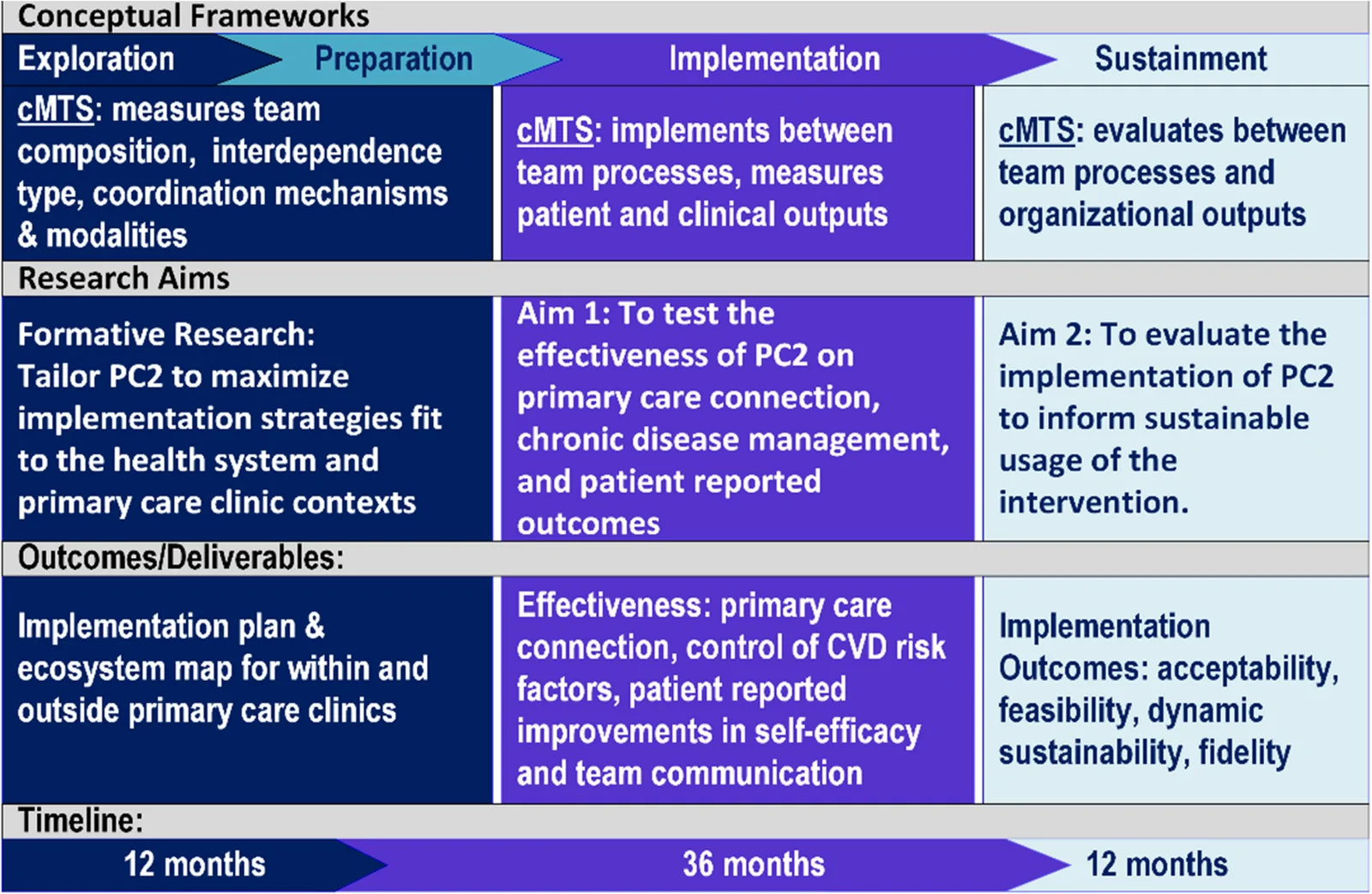
Study schema and theoretical framework

Our investigation is guided by the Exploration, Preparation, Implementation and Sustainment (EPIS) and cancer Multi-team System (cMTS) frameworks (Fig. 1). Our formative research focused on *Exploration* and *Preparation*, or the “planning” phase of implementation. To assess fit with the environment and patient needs, we conducted depth interviews with patients, cancer care providers, and primary care clinicians. Prototypes of implementation strategies were iterated in meetings with a Scientific and Cancer Survivor Advisory Council and Implementation Planning Groups, whose insights guided prototype development, iterative refinement, and interpretation of our multi-method assessment of PCC’s fit with patient need and the health system contextual realities. The trial focuses on *Implementation* and *Sustainment.*

The cMTS is the foundation of ‘shared care’ coordination for complex cancer survivors. The cMTS framework complements EPIS in important ways, providing key concepts of team inputs (e.g., team composition/complexity, boundary status) and processes (e.g., communication, mental models) and the interdependent processes within and between teams that are influenced by contextual factors that may impact PCC’s effectiveness [52]. These systems are networks of interdependent teams working toward an overarching goal (i.e. receipt of high-quality comprehensive care) with composite teams owning specific tasks and processes (e.g., cancer treatment, CVD risk management, etc.) [52–54]. A care coordination orientation in a cMTS focuses on the context, inputs, and processes of two or more interdependent but separate teams engaged in organizing patient care delivery to achieve an over-arching goal [52, 54, 55]. The constructs of the cMTS informed our formative research and are integrated into research questions in our process evaluation (Table 2). 

## Methods

### Study design

PCC will be tested in a hybrid type 3 effectiveness-implementation randomized controlled trial assessing care of 266 complex cancer survivors (*n* = 133 in PCC and *n* = 133 receiving usual care).

#### Implementation research questions & hypotheses

##### Aim 1: test the effectiveness of PCC on primary care connection, chronic disease management, and patient reported outcomes.

- RQ1: Is PCC effective in preventing a disconnect between complex survivors and their PCPs during active treatment?
- H1: PCC will yield greater primary care connection during active treatment than usual care.
- H2: PCC will improve CVD risk control among complex survivors compared to usual care.
- H3: PCC will align with care team complexity with CVD risks compared to usual care.
- H4: PCC will report higher quality communication and self-efficacy for chronic disease management compared to usual care.

#### Learning evaluation research questions

##### Aim 2: evaluate the implementation of PCC to inform sustainable usage of the intervention

- RQ1: What multi-level (patient, team, contextual) factors are associated with connected care for complex survivors (cMTS inputs and processes)?
- RQ2: How are mental models related to PCC aligned across teams (shared understanding; role clarification; fidelity)?
- RQ3: What are the similarities and differences in utility and suitability of PCC between health system affiliated and community primary care practices (acceptability; feasibility; boundary status)?
- RQ4: What innovation adaptations are needed to integrate or institutionalize core components of PCC (feasibility, sustainability)?
- RQ5: How does what is being learned within and between teams fit within the broader learning healthcare system (dynamic sustainability)?

### Participants & procedures

#### Recruitment and randomization procedures

This study will recruit from the Rutgers Cancer Institute, a National Cancer Institute (NCI) designated cancer center located in New Brunswick, New Jersey. Patients will be identified by research study staff with the assistance of cancer center tumor study groups (breast, genitourinary, gynecological) within 3 months of their initial visit to the cancer center. Research study staff will identify and invite eligible survivors to enroll in the study using multiple media such as personalized emails, mailed letters, posters with QR codes, websites, and social media campaigns. Participants will provide e-signature consent prior to enrollment.

A two-phase stratified randomization will be used, based on the strata of PCP affiliation with health system (affiliated vs. not) [56]. After verification of a patient’s unique PCP, patients will be assigned to a study arm. If a second patient is recruited who shares the same PCP as a randomized participant, the newly enrolled participant will be automatically assigned into the same treatment arm. While this is expected to be a rare occurrence, patients will be oversampled by 10% to address any implications on statistical power.

#### Patient inclusion criteria

Patient inclusion criteria for this study include patients over 18 years old who: (1) were diagnosed with any of the following cancers [Stage I-III]: breast, prostate, urinary bladder, and endometrial in the past 6 months; (2) are being treated for cancer with curative intent; (3) had their initial patient visit at the cancer center in the last 3 months; (4) have ≥ 1 CVD risk factor (hypertension, hyperlipidemia or type II diabetes) before cancer diagnosis that requires medication management; (5) are currently receiving care from a PCP; and (6), speak English or Spanish. Patients will be excluded if they: (1) had a myocardial infarction or stroke within the previous year; (2) have heart failure with an ejection fraction < 30%; (3) have stage IV-V chronic kidney disease (eGFR < 30); or do not speak English or Spanish. Patients whose cancer progresses to metastatic disease during the trial will be allowed to continue to participate unless they voluntarily withdraw.

#### Health care team inclusion criteria

Health care team participants will be drawn from both oncology and primary care settings.

Cancer care team members affiliated with Rutgers Cancer Institute will be invited if they are actively involved in the treatment or care coordination of an enrolled patient and a member of the participating tumor study groups. Primary care clinicians will be recruited based on their existing relationship as the established PCP of an enrolled patient. Both health system-affiliated and unaffiliated/community-based practices will be invited to participate.

### Primary care connect: health system intervention & implementation plan

Survivors randomized to usual care will receive nurse navigation as directed by their care team (which routinely advises patients to identify a PCP) and a referral to the Rutgers Cancer Institute Resource Center. PCC is a suite of multi-level implementation strategies to build primary and oncology team capacity, standardize care communication protocols between care teams, and prime complex cancer survivors about the need for shared care starting at cancer diagnosis (Table 1).

**Table 1:** Primary care connect intervention components, implementation strategies and corresponding evaluation measures

Level	Components	Actors	Actions	Action Target	Timing	Implementation Evaluation	Justification
Patient	Patient Explainer Video & Educational Packet	RA Patient	RA shows patient the explainer video RA distributes educational materials (in-person & virtually)	Patient education & self-efficacy to manage chronic conditions	BL visit	Acceptability	Increase knowledge about need for CCM during cancer treatment Empower patients about the action steps they can take to improve CCM (self-efficacy)
Patient	Receipt of Care Plan	RA Patient	Received through portal at 6 weeks	Patient self-management	< 6 weeks post BL	Acceptability Appropriateness	Provide patient with information to use with PCP & any other specialists involved with CCM
CCT	Training/In-reach	PIs CCT	Training about intervention & Care Plan Process	Front line staff knowledge & confidence in intervention	1–2 weeks prior to BL	Feasibility of training Acceptability of planned workflow Adaptations of process	Increase understanding of modified clinical workflow and reduce fragmented care
CCT	Care Plan Production	RA CCT	RA drafts Care Plan & sends to CCT CCT suggests edits & approves for use RA sends Care Plan to PCP & Patient	Between team Communication	Transmission to PCP within 6 weeks of BL visit	Feasibility Adaptions of process Acceptability of clinical workflow Fidelity	Increase knowledge of PCPs about cancer plan & potential impacts on CCM Clarify roles of different teams—PCP & CCT
PCP Team	PCP Explainer Video &-Welcome Packet	RA PCP Team	RA emails or mails welcome packet	Orientation to intervention: role clarity	< 6 weeks post BL	Acceptability Appropriateness	Standardize the mental model for role clarity Reduce information overload
PCP Team	Receipt of Shared Patients’ Care Plan	RA PCP Team	RA transmits plan to PCP team	Knowledge transfer PCP confidence	< 6 weeks post BL	Adoption Acceptability Appropriateness	Implement a secure, asynchronous communication protocol to ensure PCPs receive actionable information
PCP Team	Ambassador Program	RA PIs Cancer Care Contacts	RA transmits info with welcome packet; creates direct access channels to CCT	Centralized communication hub Real-time troubleshooting	< 6 weeks post BL; quarterly outreach	Acceptability Feasibility	Create a low cognitive load pathway to address clinical questions and reduce navigation fatigue
Health System	EHR Optimization	Informatics team	Optimize care plan production efficiency	Use emerging tech to streamline care plan development	Ongoing	Feasibility Adaptations	Increase usability of technology to develop care plan to promote sustainability
Health System	EHR Optimization	RA	EHR accuracy of clinical team members	Communication efficiency	Baseline	Feasibility	Ensure accurate clinicians listed for internal communication

*RA *Research Assistant, *BL *Baseline, *PCP *Primary Care Physician, *CCT *Cancer Care Team, *CCM *Chronic condition management, *EHR *Electronic health record

#### Complex cancer survivor educational materials

Chronic care models for CVD risks and cancer emphasize psychoeducational approaches to empower patients to participate in conversations and goal setting with their care teams [57]. Survivors enrolled in PCC will receive usual care supplemented by an educational program about shared care, available in print and electronically. These educational materials will include an “explainer video” which is a 3-minute orientation video that describes why remaining connected to primary care is important for chronic disease management. This is supplemented with virtual and print materials that include messaging about the need for routine chronic disease management visits, taking prescribed medications as directed, and communicating with their care team members when problems arise.

#### Templated care coordination communications

We will use templated, EHR-based correspondence (referred to as ‘care plans’) from the cancer team to the primary care team at 6 weeks post-enrollment and 10 months. The initial communication will include the patient’s cancer diagnosis/treatment, goals of treatment, impact of treatments on CVD based on Healthcare Effectiveness Data and Information Set (HEDIS) quality measures [58–60], and a recent state-of-the art clinical review [61]. Patients will also receive a copy of these care plans and instructions on how to use them with primary care or other subspecialists to communicate key information about their cancer care.

#### Cancer care teams: intervention implementation and training

Educational sessions will be hosted with each tumor study group (breast, gynecological, and genitourinary) to orient the cancer team about PCC. In-reach will include educational messages about the role of primary care, optimal comorbidity management with primary care engagement, the need for clear communication pathways, and delineation of the roles for cancer and primary care. As PCC was designed specifically to improve between-team communications, we will provide instruction about the best mode for communication with internal PCPs (e.g., chat function within Epic), responsiveness to PCP inquiries (e.g., timeliness, clarity), and guidance to foster shared understandings between the teams. We will also be reviewing their role in reviewing and approving the templated care communication documents for patients enrolled in the PCC arm of the trial.

#### Primary care teams: ambassador program

PCPs of complex cancer survivors in the PCC arm will receive outreach. Primary care team members will be invited to join the Primary Care Ambassadors Program, which will send brief communication about key survivorship clinical issues (e.g., information needs, psychosocial care, cardio-oncology and survivorship). Primary care teams will receive welcome kits containing materials about the importance of shared care for complex cancer survivors, including the link to an explainer video. In these kits, we will invite the patient’s PCP to join the Ambassadors Program to facilitate information flow between cancer and primary care teams. We will communicate that PCC components were designed specifically to address ongoing primary care challenges and to foster closed loop communications by providing clear and concise information about the impact of cancer treatments on CVD risks, the PCP’s optimal role in shared care, and whom to contact and when to reach out to the cancer team.

#### Health system: EHR optimization

At the time of patient enrollment, the research assistant will review the members assigned to a patient’s care team to ensure accuracy. Throughout the trial, the health system informatics team will continue to refine the procedures to optimize the efficiency for templated care coordination communications. We will continually refine the procedure as emerging technologies are integrated into care delivery.

### Data collection

Patient self-report and clinical assessments of CVD control will be collected at baseline, 6-, 12-, and 18-months. See Table 2 for the list of data constructs, measures, and collection strategies. As this is a minimal risk study, a formal data monitoring committee is not warranted. We will utilize standard institutional procedures and infrastructure for data safety and monitoring to ensure security of participant data.

**Table 2:** Data constructs, measures, and collection strategies

Construct (s)	Measures	Collection Strategies	Timepoint			
Main Outcomes			BL	6 mo.	12 mo.	18 mo.
Connected Care	Author adapted scales	Patient Survey	X	X	X	X
CVD risk control	glycated hemoglobin [A1c], lipid profile, blood pressure	Lab testing Office BP	X	X	X	X
Patient self-efficacy	PROMIS Self-efficacy for Managing Chronic Conditions [62]	Patient Survey	X	X	X	X
Patient-provider communication	Patient-centered communication scale [63]	Patient Survey	X	X	X	X
Potential Moderators						
Demographics	Biological factors (sex, age, race/ethnicity), education, health insurance	Patient Survey	X			X
Health & Cancer-Related Factors						
Cancer diagnosis	n/a	Chart Audit	X			
Cancer treatment	n/a	Chart Audit	X			
Cancer related Distress	NCCN Thermometer [64]	Chart Audit	X			
Psychosocial Illness Impact (-/+)	PROMIS Psychosocial Illness Impact postive/negative SFs [65]	Patient Survey	X		X	
Cognitive Function	PROMIS SF 6 [66]	Patient Survey	X		X	
Pain interference	PROMIS-Cancer Pain Interference [67]	Patient Survey	X		X	
Fatigue	PROMIS Cancer Fatigue SF [68]	Patient Survey	X		X	
Charlson Comorbidity Index	n/a	Chart Audit	X			X
Lifestyle/Behavioral Factors						
Alcohol use	PROMIS SF [7]	Patient Survey	X		X	
Smoking history/use	n/a	Chart Audit	X			X
Medication Adherence	Adherence to Refills and Medications scale (ARMS) [69] and Self Efficacy for Appropriate Medication Use Scale (SEAMS) [70]	Patient Survey	X	X	X	X
Obesity Status	Body mass index	Chart Audit	X			X
Patient Experience						
Patient-centeredness	Patient-centered survivorship index [71]	Patient Survey	X	X	X	X
Care team composition/complexity	Author adapted scales	Patient Survey	X	X	X	X
Medical mistrust	Group-based medical mistrust scale [72]	Patient Survey	X		X	
Implementation Outcomes						
Intervention Appropriateness	Intervention Appropriateness Measure (IAM) [73] Author adopted scales	PCP Team Survey CCT Team Survey	X		X	
	Author created semi-structured interviews & observations	Key Informant Care Team Interviews Post-intervention Care Team Fieldnotes/ Implementation Tracking			X	X
Acceptability	Acceptability of Intervention Measure (AIM) [73] Author adopted scales	Patient Survey PCP Team Survey CCT Team Survey Key Informant Care Team Interviews	X		X	
	Semi-structured interviews and observations	Team Interviews Post-intervention Care Team Post-intervention Patient Interviews Fieldnotes/ Implementation Tracking			X	X
Feasibility/Adaptations	Feasibility of Intervention Measure (FIM) [73] Author adapted scales	PCP Team Survey CCT Team Survey	X		X	
	Semi-structured interviews and observations	Key Informant Care Team Interviews Post-intervention Care Team Interviews Post-intervention Patient Interviews Fieldnotes/ Implementation Tracking				
Dynamic Sustainability	Author adapted scales	PCP Team Survey CCT Team Survey			X	
	Semi-structured interviews and observations	Key Informant Care Team Interviews Post-intervention Care Team Fieldnotes/ Implementation Tracking			X	X

#### Primary outcome: connected care

Patient surveys administered at baseline, 6-, 12- and 18-months will assess *timeliness* of connected care (having 1 chronic disease focused visit within the first 6 months after enrollment) and *ongoing connected care* (having 3 chronic disease focused visits during the 18-months study period). Additionally, assessment will include: (1) when primary care visits occur and the reason for the visit; (2) the format (virtual or in office); and (3) the timing of primary care visits. 

#### Secondary outcome: control of CVD risk factors

HEDIS quality measures will be used to assess CVD risk control using clinical and laboratory tests ascertained during research visits at baseline, 6-, 12- and 18-months. HEDIS criteria are as follows: blood pressure < 140/90 mm Hg; glycated hemoglobin A1c < 8.0%; on statin if patient has diabetes, cardiovascular disease, LDL *≥* 190 mg/dL, or 10-year estimated ASCVD risk ≥ 10% [58–60]. Some patients may have more than one CVD risk to manage; therefore, CVD will be a continuous variable calculated by the percent of criteria met based on individual patient comorbidity burden. 

#### Tertiary outcomes: self-efficacy for managing chronic conditions

Patient-Reported Outcomes Measurement Information System (PROMIS) measures will be used to assess an individual’s confidence in his/her ability to successfully perform specific tasks or behaviors related to one’s health in a variety of situations assessing five domains: daily activities, symptoms, medications and treatments, emotions and social interactions [62]. *Patient-centered communication* that focuses on the patient’s perspectives and values and supports shared decision-making [74–77] with both the cancer and primary care team will be assessed using a validated 7-item scale that utilizes concepts from Epstein and Street [77] to probe experiences with their providers. We will adapt this scale slightly to use a 6-month recall period given the intensity of care during the active cancer and our planned assessment intervals. 

#### Sociodemographic variables and potential moderators

All sociodemographic variables and potential moderators will be assessed using self-reported items from the patient survey (except CVD risks, comorbidity, and obesity). A multi-measure approach for medication adherence will be used as this has greater sensitivity than relying on a single self-reported measure. This approach will include two validated scales—Adherence to Refills and Medications scale (ARMS) and the Self Efficacy for Appropriate Medication Use Scale (SEAMS)—as both have strong validity and reliability among several chronic conditions that require medication management [69, 70, 78]. This approach is necessary because objective measures are not feasible for complex cancer survivors receiving primary care in unaffiliated practices [79]. Patient-centeredness will be measured with the patient-centered survivorship index, which includes seven factors: (1) information and support; (2) having a medical home; (3) patient engagement in care; (4) care coordination; (5) insurance navigation; (6) care transitions from PC to oncology; and (7) prevention and wellness services [71].

### Data analysis

The proportion of individuals with at least one visit within 6 months prior to follow-up will be compared for PCC versus usual care via a z-test. In the case that there are instances of more than one individual per primary care practice, a multiple imputation approach will create multiple data sets with one individual randomly selected per practice and results combined by evaluating variance between and within data sets. The same approach will be used to evaluate PCC impacts whether someone has three visits within 18 months. For the secondary outcome, descriptive statistics and graphs will summarize the distribution of averages of percent of criteria met, both overall and by treatment group and time. Percent of criteria met for CVD risk factors will be treated as a continuous variable with t-tests to compare means between PCC versus usual care. Tertiary outcomes will be evaluated similarly. After theoretical models are used to identify potential modifying variables, exploratory analyses will use linear models with interactions between treatment arm and will be used to assess each potential modifier. Theoretically selected mediators will also be evaluated using structural equation modeling. Missing data will be assessed for patterns and appropriate statistical methods will be employed to assess impact of missingness on trial outcomes. 

#### Power calculations

Power is calculated assuming comparison of two groups using z-tests for differences of proportions at the 0.05 significance level. If we suppose that for the control group, the percentage of those with at least one primary care visit is 50%, then we would be able to detect if 70% of those within the PCC group had at least one visit with 90% power if each group has 121 subjects (242 total). Even with only 104 subjects (14% reduction in sample size due to either loss to follow-up or other data issues), we have 85% to detect an effect. We are oversampling by 10% (total *n* = 266) to account for instances when survivors share the same primary care practice team and need to be similarly assigned to a treatment arm. We expect to be able to recruit at least 121 total patients per arm. In the last year, our cancer center had 589 new patients with the target cancers (bladder, breast, prostate, endometrial).

Experience with this population suggests that 15% of these came for second opinions, leaving approximately 501 who presented for care. Thus, we would need less than 50% recruitment success among cancer patients presenting to CINJ to reach our target number of patients.

#### Learning evaluation data collection

The EPIS domains of *Implementation* and *Sustainment* and selected constructs from cMTS will inform a learning evaluation strategy to assess implementation processes. This evaluation allows for insights about adaptions and opportunities to optimize adoption of the intervention [80, 81]. Using implementation process data, depth interviews, and primary care and oncology care team surveys, we will compare similarities and differences between primary care engagement with PCC based on health system affiliation (affiliated PC vs. non-affiliated). We will also compare key domains for the cMTS for affiliated and non-affiliated primary care and oncology team processes.

Implementation process data that include qualitative observations and key informant and depth interviews will be used to understand: (1) the cancer team dynamics and environment in which the intervention is initiated; (2) contextual features that enhance or inhibit adoption of the components of the intervention; (3) any adaptations to the intervention to conform to contextual needs; and (4) how the intervention was implemented. Patient surveys will be used to track MTS team complexity with care team changes and visit information collected at baseline, and 6-month, 12-, and 18-month time points.

Patient interviews will be conducted with selected patients after the 18-month survey is completed.

These interviews will use a modified multi-moment timeline interview grounded in sensemaking methodology to gain insights into how patients make sense of who is on their team, the team’s complexity, and changes that occurred to care team composition during their cancer care [82]. This approach seeks to identify gaps encountered by patients related to communication between their care teams, the bridges they built to overcome those gaps, and the outcomes of the information exchanged to evaluate how actors in the cMTS interpret and adapt to the intervention in real-time.

Process data of cross-team communications will include a standard template for the functional pieces of operationalizing the templated care plan that will include within-team timeframes for review and approval, technology issues, and research assistant time on task. An evaluation team member will debrief with the cancer teams every 3 months to discuss the process of implementation and to document any patient, team or system issues that have arisen and any adaptations made to enhance the flow and efficiency of the intervention. Fieldnotes will be prepared to record these key informant interviews and attend to how contextual factors (e.g., physical space; organizational, clinician or patient features; care team workflows or processes) impact implementation efforts. Attention will be paid to contextual features and interactions that were indicative of positive or negative alignments between intervention and local setting and instances where better alignments serve to enhance implementation progress (e.g., “implementation measures” as outlined in EPIS). Observational data will be captured in detailed fieldnotes and written up quarterly as case summaries for each cancer team and shared with our Advisory Council for review and input.

The research assistant will develop and update an ongoing care pathway map for complex cancer survivors tracking who is involved with each patient’s care over time, assessing the complexity of the multi-team system. Chart audits and patient surveys will be used to update each patient’s care map that will visually display care team members and critical events (e.g., cancer therapy dose reductions, CVD-related hospitalizations).

Online process evaluation documents for the templated care plans will be completed by research assistant and serve as a real-time communication between the implementation and evaluation teams [83].

Research assistants will input critical incidents, communications from care teams, and reflective process notes about the templated communication process. Brief entries will be captured in REDCap and include patient ID, team members involved, successes and failures of communications. During key informant interviews, evaluation team members will elicit additional information about entries as needed. These will capture what issues arise when sharing patients with primary care clinics within and outside the health system. 

##### Depth interview sampling and recruitment

At the 12-month intervention timepoint for our learning evaluation, we will recruit a purposive sample (*n* = 20) primary care team members stratified by primary care setting type: (i) Rutgers Robert Wood Johnson Barnabas Health (RWJBH) system-affiliated practices and (ii) unaffiliated practices. With patient’s permission, we will acknowledge shared patients’ consent and enrollment in the trial. The learning evaluation will also include patients (*n* = 20) after completion of the 18-month survey. We will sample primary care teams and complex cancer survivors using a maximum variation sampling strategy. Process evaluation insights will guide case selection addressing any logistical and clinical issues that emerged during implementation.

##### Depth interview data constructs

The care pathway maps will be used to guide depth interviews to assess key cMTS constructs from the patient, cancer care team member, and primary care team members perspectives. cMTS “process” constructs will be assessed: collective sensemaking, team leadership, trust, mental models, cooperation, and psychological safety. We assess primary care clinic coordination mechanisms (e.g., tools and strategies used to sequence and align interdependent care) [52] and the compatibility of PCC with the clinic coordination modalities. 

##### Team-based survey recruitment

Primary care teams that have a patient enrolled in ACCESS-PC and any team member identified in the Ambassadors Program will receive an email invitation and/or mailed survey invitation packet. We estimate a total of PCPs (*n* = 266), half in the intervention arm (*n* = 133) and the other half in the control arm (*n* = 133). In the Rutgers Cancer Institute, teams are organized by tumor study group (e.g., breast, genitourinary, gynecological) and treatment modality (infusion center, radiation oncology) (*n* = 75). We will invite members of the tumor study groups, infusion center, and radiation oncology teams involved in each patient’s care. We will use methods associated with better physician response rate and anticipate ~ 55% response rate [84–86]. 

##### Primary and cancer care team data constructs

We will assess inner- and outer- practice characteristics of the primary care sites including implementation climate, payor mix, and practice site type in a module completed by PCPs (as this will be consistent in both the control/intervention teams). We will adapt subscales of the Survey of Physician Attitudes Regarding the Care of Cancer Survivors (SPARCCS) to assess cross-team attitudes, awareness, and practice patterns for: preventive care, coordination of care, preferences for follow-up care models and role perceptions [84]. Author adapted scales will be used in the team survey to assess all participants’ experiences and perceptions about the implementation strategies and key within and between team attributes [87]. We will assess within and between team assessments of having shared superordinate goals because this is a key characteristic of multiple teams functioning as an interdependent cancer multi-team system (Table 2). 

##### Implementation constructs

We will assess four core implementation outcomes: (1) *Appropriateness* using the Intervention Appropriateness Measures (IAM) and key informant interviews and fieldnotes [73]; (2) *Acceptability* using the Acceptability of Intervention Measures (AIM) and the post-intervention interviews with care team members and patients [73]; (3) *Feasibility/Adaptations* will be measured via the Feasibility of Intervention Measures (FIM), implementation tracking, interviews with care team members and patients, and fieldnotes [73]; (4) *Dynamic Sustainability* [88] will be assessed using author-adapted scales, process evaluation data, case summaries, and depth interviews with key actors in the health system to determine if the intervention can evolve and adapt within evolving health system constraints.

#### Analysis of learning evaluation

##### Qualitative analysis

Ongoing analyses of data collected by our evaluation team will be fed back to the intervention team to inform them of progress, areas in need of contextual alignment, and opportunities for further adaptation. On a rolling basis, we will analyze cancer survivor pathway, implementation process data and interviews using a comparative case analysis. As data are analyzed, a working summary of emergent findings will be maintained and continuously updated as incoming data are added to the project. Intervention baseline, 6-month and 12-month quantitative and qualitative results will be summarized in brief reports with recommendations to be shared with Advisory Council members for reflections on any changes needed. During the 12 months of observing intervention and sustainment activities, we will also integrate collected data to conduct theory-driven, research-focused analyses for broader dissemination and publication purposes. These mixed methods analyses will focus on patterns of relationships between the clinical effectiveness and implementation outcomes. 

##### Quantitative analysis

Descriptive statistics and graphs will summarize the distribution of team-based outcomes overall as well as by treatment group and time. For comparison of responses from staff from PCC versus usual care practices, the multiple responses from the PCC practices will be averaged together so that each practice has one measured value for each outcome. Mixed models, with a random effect for practice, will use an interaction term between indicator variables for treatment arm and time to assess the effect of treatment on changes over time. Covariates for characteristics such as size of practice will be included to control for confounding and, in exploratory analyses, study modification of the treatment effect.

For patients, measures from chart audits will be compared across all time points. Binary outcomes will be assessed via hierarchical logistic regression models that include random effects for practice, indicator variables for time and treatment, as well as interactions between time and treatment. Interactions will be tested via F-tests for effect of the intervention estimated across time points. For continuous outcomes from patients, such as percent of criteria met for CVD risk factors, a similar approach will be taken with mixed linear models such that there is a random effect for person. In exploratory analyses, factors that identify subsets of practices will be identified and considered as modifiers by looking at the interaction between time, treatment arm, and team characteristics. 

##### Integration and synthesis of quantitative and qualitative findings

We will integrate qualitative and quantitative findings using joint displays, an integration strategy that brings together quantitative and qualitative data to illustrate insights around effectiveness and implementation outcomes [89]. These can be organized by conceptual framework to illustrate the effect of an intervention at multiple levels. Integrated findings will be disseminated to primary care teams in a newsletter format to promote engagement and ensure our findings are locally disseminated.

## Discussion

The overarching goal of this study is to examine whether cancer survivors becoming “lost in transition” is a preventable problem. Our health system intervention tests whether the challenge of transitioning survivors back to primary care can be avoided altogether by re-engineering care coordination with primary care at the outset of cancer treatment. When cancer survivors have a comorbid condition at diagnosis, it presents a challenge to both the individual patient and the care delivery system to coordinate care across teams and settings [10, 15, 90]. Hypertension, diabetes, and hyperlipidemia have strong, well-established evidence based guidelines for CVD risk management; however, cancer survivors often do not receive this necessary shared care during cancer treatment [91–95]. This study aims to translate the recent state-of-the-art clinical guidelines for CVD risk management in cancer survivors [61] through capacity building at the patient, primary care provider, and oncology team and health system levels.

Majority of care coordination interventions test single strategies and rarely account for context [96]. Static approaches, like survivorship care plans and guidelines for primary care, that lack attention to the implementation context and care delivery processes have proven insufficient to shift care paradigms and produce shared care for complex cancer survivors [40, 97]. Therefore, research such as this study foregrounds the realities of primary care to create sustainable coordination processes and accelerate the adoption of shared care [97, 98].

PCC addresses these critical gaps in the broader care coordination and cancer team-based care empirical evidence bases [35, 36, 96]. First, while less than 6% of cancer care coordination research is guided by theory, our research plan is guided by integrated health care delivery and implementation science frameworks aiming to understand the dynamics of imposing new care delivery processes from multiple health system perspectives (e.g., complex cancer survivors, primary care, and cancer care team members) [35]. Second, while multi-level interventions are rare in the cancer care coordination literature, we propose mixed methods multi-level evaluations of PCC to provide a comprehensive understanding of the alignments or disconnections between cancer and primary care. Third, a review of team-based care for cancer survivors with comorbidities found 4 studies that focus on “shared care,” with only one focused on CVD risks and none explicitly measuring teamwork processes [36]. Our approach addresses these issues explicitly with methods like health system mapping [99] and prioritizing contextual assessment. As multi-level and contextual factors can facilitate or impede care coordination [42, 43], PCC will be tailored and tested in a hub-and-spoke model to systematically evaluate such factors.

A critical innovation of this study is the focus on composition and complexity of a patient’s care team. Previous research on cMTS in newly diagnosed cancer survivors with cardiopulmonary disease and diabetes reported that oncology and primary care (37%) was the most common cMTS composition [100]. In the same study, high cMTS complexity (defined as ≥ 4 clinicians from ≥ 2 specialties) was lower for non-Hispanic Black and Hispanic survivors than for Non-Hispanic Whites, and for Medicaid-Medicare beneficiaries compared to survivors with Medicare only [100]. Therefore, this study specifically examines cMTS composition and complexity for each patient as a mechanism that may impact care quality.

## Conclusion

Cancer care is inherently complex. Cancer treatment is multi-modal and delivered at multiple organizational levels, by multiple providers on different teams, who may be geographically dispersed and may span health system boundaries. This study utilizes a hybrid type III effectiveness-implementation design to test a multi-level, multi-component health system intervention and a mixed methods evaluation to understand the influence of context, inputs, and processes on implementation.

## Supplementary Information

Supplementary Material 1.

## Data Availability

The quantitative datasets generated and/or analyzed during the study will be available in a generalist repository upon completion of the study. The qualitative datasets will not be publicly available due to privacy concerns but are available from the corresponding author on reasonable request.
